# Success of Penile Replantation Using Combination of Cialis, Hyperbaric Oxygen, and SPY Technology

**Published:** 2019-02-05

**Authors:** Karen B. Lu, Kyle Sanneic, Jeffrey A. Stone, Allen Morey, Bardia Amirlak

**Affiliations:** ^a^University of Texas Southwestern Medical Center, Dallas, Tex; ^b^Institute for Exercise and Environmental Health, Texas Health Dallas

**Keywords:** penile amputation, penile replant, hyperbaric oxygen, Cialis, indocyanine green dye

## Abstract

**Objective:** There are very few studies reporting the techniques utilized in penile replantation. Of those in literature, many agree that the use of microvascular technique results in better outcomes. The most common complications are skin necrosis and venous congestion, which are even higher in replants without arterial supply. **Methods:** This study describes a case of self-inflicted penile amputation treated with microsurgical replantation and managed postoperatively with hyperbaric oxygen therapy and Cialis (tadalafil), and SPY angiography. The penile replant had extensive skin necrosis, which prevented a sufficient clinical evaluation of the replanted penis. Serial SPY angiography was performed to assess tissue viability, following hyperbaric oxygen therapy and Cialis treatment. **Results:** SPY angiography was critical to the decision making of the operating team in the management of this case of penile replantation. **Conclusions:** The use of SPY angiography prevented the patient from undergoing revision amputation and allowed for a safe and successful penile replantation.

Penile amputation is a rare event, often inflicted due to automutilation, psychosis, or trauma.^1^ Because of the limited amount of cases, there is a paucity of literature regarding penile replantation. However, of the cases described, microsurgical technique seems to be the preferred method for penile replantation.^2^^-^4 Microsurgery allows for maximal tissue preservation and lower risk of complications.^5^^,^^6^ And as with many microsurgical procedures, the most common complications following surgery are skin necrosis and venous congestion, due to a lack of adequate perfusion.^1^ Survival in a replanted penis without microvascular repair requires adequate sinusoidal blood flow and is associated with an even higher incidence of skin necrosis, fistula formation, urethral stricture, permanent loss of sensation, and impotence.^7^

The use of SPY Elite laser angiographic system (Lifecell, Inc/Novadaq Technologies, Concord, Ontario, Canada) has been heavily used by the senior author in guiding intraoperative decision making and intimately interwoven with our algorithm for tissue ischemia.^8^ This article introduces the use of SPY angiography in the management of penile replantation following decreased tissue perfusion and skin necrosis. Being that tissue perfusion is paramount in the survival of the replanted penis, functionally and cosmetically, the use of SPY greatly enhances the clinical outcomes of replantation and provides better insight to tissue survival in a cosmetically sensitive area. The use of SPY angiography with indocyanine green increases predictability of tissue viability, allows for objective decision-making, and increases patient safety.

## METHODS

**Figure 1:**
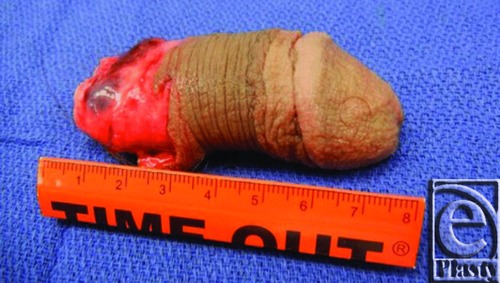
Showing an amputated penis. Distal amputated penis, 7.5 cm in length, was transected 2 cm from the base of the penis.

**Figure 2:**
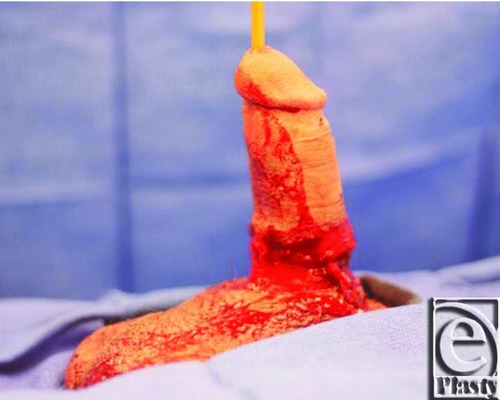
Figure after completed surgery. Following repair of 2 dorsal veins and no artery anastomosis, the replanted penis showed excellent perfusion with bright red bleeding and no venous congestion.

A 22-year-old man, with a history of depression, was transferred to our facility after sustaining a self-inflicted complete transverse penile amputation. The transection was 2 cm from the base of the penile shaft, and the distal amputated part was preserved appropriately. (Figure 1 shows the length of amputated penis.) The incident had occurred 16 hours prior to operation time. The patient requested penile replantation and proper consent was obtained. The repair of the urethera, corpus cavernosum, and corpus spongiosum was performed by the urology team. A Foley catheter and suprapubic catheter were inserted, and proximal and distal ends of the penis were approximated and prepared for microvascular surgery. Two large caliber deep dorsal veins were identified and repaired with a 3-cm vein graft from the right forearm. Throughout the procedure, there was evidence of adequate perfusion to the distal aspect of the penis, apparent by bright red bleeding from the skin margins as well as venous oozing from the deep dorsal veins. Given the evidence of excellent perfusion of the distal amputated part and lack of identifiable arteries for anastomosis, an arterial anastomosis was not performed. The dorsal nerves were then isolated, proximally and distally, and coapted using interrupted nylon sutures. Buck's fascia was repaired, and then the skin on the ventral aspect of the penile shaft was reapproximated. Of note, a Cook Doppler over the repaired veins showed no signal, even though there was clinical evidence of blood flow through the veins. A transcutaneous Doppler did not detect any signal over the veins as well. At the completion of the procedure, there was bright red bleeding noted from the glans penis, and no evidence of venous congestion. (Figure 2 shows bright red bleeding after the surgery.)

**Figure 3:**
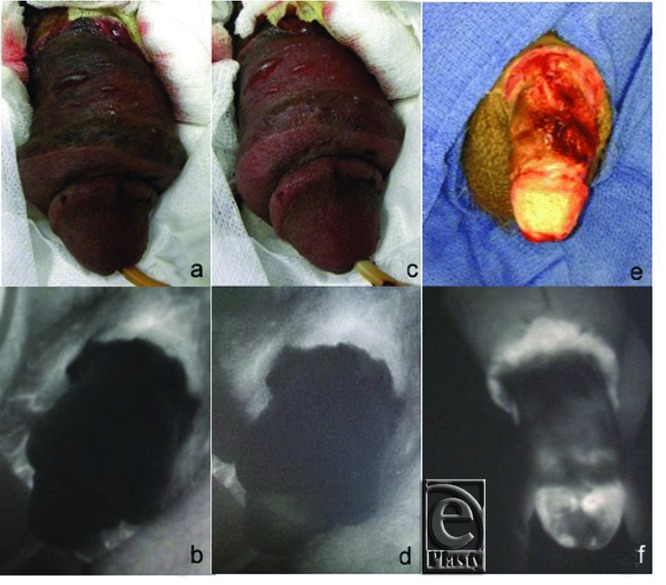
Figure of SPY angiography imaging and standard photos of the replanted penis. Standard clinical evaluation is not sufficient to adequately judge the level of perfusion at the distal penis. (*a*) Prior to HBOT and Cialis treatment, clinical evaluation was extremely difficult. (*b*) SPY angiography during initial clinical evaluation showed poor perfusion. (*c*) After initial treatment with HBOT and Cialis, it was difficult to clinically appreciate any difference. (*d*) With SPY angiography, perfusion at the distal tip of the penis, after initial treatment with HBOT and Cialis, is appreciated. (*e*) Standard photography after debridement of tissue showing healthy tissue and good perfusion. (*f*) SPY angiography image of the penis after debridement shows excellent perfusion to the tip.

Shortly after surgery, the replant began to show evidence of decreased perfusion. The skin around the shaft turned from dusky to dark black and became thickened, making clinical evaluation extremely difficult. The glans penis had similar color appearance but was soft with no evidence of congestion. Clinical evaluation in the form of doppler, needle prick, and physician examination was not sufficient to produce any answers. Poor tissue perfusion was confirmed by SPY angiography. A national shortage of topical nitroglycerin limited the interventions for salvage with this drug. Cialis (Lilly, Indianapolis, Indiana) and hyperbaric oxygen therapy (HBOT) were initiated 72 hours after amputation and 58 hours after replantation. Due to the fact that this replant had no arterial anastomoses, HBOT was additionally vital as it would help increase blood flow to the penis. After HBOT and Cialis, physical examination still yielded no answers but repeat SPY angiography showed increased tissue perfusion at the distal tip of the glans which prompted continuation of the Cialis and HBOT. (Figure 1 and 3 show SPY angiography prior to HBOT and Cialis treatment, after HBOT and Cialis treatment, and after debridement). The skin around the shaft of the penis became necrotic, which was expected. Two weeks after the initial surgery, the necrosed tissue was debrided. Three weeks after surgery, a split thickness skin grafting was performed.

## RESULTS

Six months postoperatively, the patient was able to achieve a spontaneous erection and engage in sexual intercourse. SPY angiography effectively demonstrated the success of Cialis and HBO therapy and prevented the patient from returning to the operating room or having to undergo amputation of his penis, due to the dead appearance on physical exam.

## DISCUSSION

The goals of a penile replantation are to regain urinary function, engage in penetrative sexual intercourse, and achieve a satisfactory cosmetic appearance.^9^ Previous literature indicates that microvascular repair, specifically venous outflow, yields superior outcomes.^1^^,^^2^^,^^10^ In regard to the number of vessels reanastomosed, there does not seem to be a consensus regarding its effective on patient outcomes.^1^

In this particular case, long ischemia time (16 hours) and lack of identifiable arteries possibly lead to a higher risk of skin necrosis. Chou et al^4^ showed that blood flow from corpus tissue was sufficient for long-term shaft and glans survival, as was the result from this case. Chou et al also described the use of HBOT to successfully treat tissue ischemia.^4^ However, Cialis has never been described in the treatment of penile replantation survival. The use of Cialis in combination with HBOT allowed perfusion of the distal amputated penis and successful replantation.

SPY angiography allowed the operating team to objectively assess the improvements made with HBOT and Cialis and justified the extensive and aggressive treatment with HBOT. Without the use of SPY angiography, the complete full-thickness necrosis of the penis, which provided an extremely difficult evaluation, would have probably resulted in a reoperation or phallic reconstruction. SPY angiography allowed for a reliable and objective method of assessing tissue viability, preventing reoperation, and allowing the team to take a more conservative approach, thereby increasing patient safety.

Successful penile replantation is critical to patients both physically and mentally. Microvascular surgical repair is the preferred method; however, the lack of an arterial anastomosis can still result in a successful replantation. The use of SPY in the immediate postoperative period helped manage complications and reduced the need to make management decisions based on insufficient clinical evidence. SPY angiography provided reliable objective data regarding tissue perfusion, after hyperbaric oxygen and Cialis therapy, and prevented the patient from undergoing unnecessary revision amputation. The ability to continually evaluate the replantation enabled the operating team to manage the skin necrosis conservative and ultimately resulted in a functional and cosmetically well-appearing penis.

## Figures and Tables

**Figure 4:**
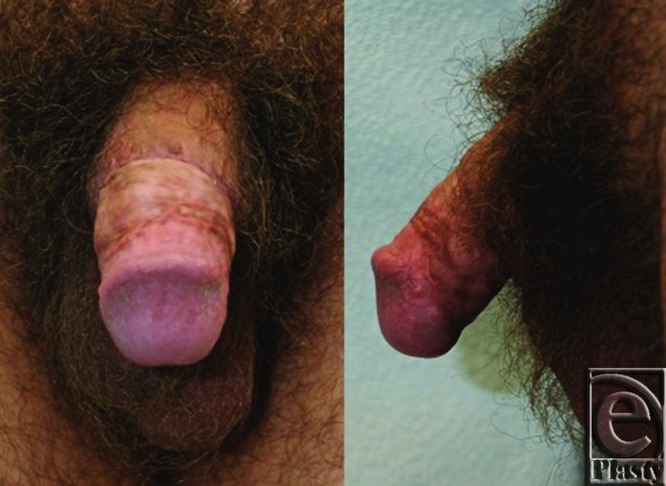
Figure showing full recovery. Six months after initial injury, patient displays full recovery with good urination, sensation, and erection.
